# Small but Mighty: Low Bio-Accessibility Preserves Polyphenols from Mini Purple Carrots for Direct Action Against Colon Cancer Cells

**DOI:** 10.3390/antiox15010113

**Published:** 2026-01-15

**Authors:** Amel Hamdi, Emel Hasan Yusuf, Rocío Rodríguez-Arcos, Ana Jiménez-Araujo, Paulina Nowicka, Rafael Guillén-Bejarano, Sara Jaramillo-Carmona

**Affiliations:** 1 Phytochemicals and Food Quality Group, Instituto de la Grasa (CSIC), 41013 Seville, Spain; ahamdi@ig.csic.es (A.H.); rrodrig@ig.csic.es (R.R.-A.); araujo@ig.csic.es (A.J.-A.); 2 Department of Fruit, Vegetable and Plant Nutraceutical Technology, The Wrocław University of Environmental and Life Sciences, 37 Chełmońskiego Street, 51-630 Wrocław, Poland; emls222224f@gmail.com (E.H.Y.); paulina.nowicka@upwr.edu.pl (P.N.); 3 Laboratory of Cellular and Molecular Nutrition, Instituto de la Grasa (CSIC), 41013 Seville, Spain

**Keywords:** carrots, biodiversity, phytochemical profile, polyphenols, bioaccessibility, antiproliferative activity, colorectal cancer prevention, functional foods

## Abstract

Carrots are exceptional sources of bioactive compounds with potential health benefits. This study investigated the relationship between the biodiversity of carrot cultivars (colour and size) and their potential chemopreventive properties. Four distinct carrot cultivars (orange, white, yellow, and purple) of normal and miniature sizes were comprehensively analysed for polyphenolic composition, bio-accessibility through in vitro simulated digestion, and in vitro antiproliferative activity against the HCT-116 colon cancer cell line. Our findings revealed that vegetable size influenced phytochemical composition more than vegetable colour, with mini purple carrots exhibiting exceptionally high polyphenolic concentrations and superior antiproliferative activity compared to orange, yellow, or white varieties. Notably, the bioaccessibility of bioactive compounds remained remarkably low across all samples, suggesting that these phytochemicals reach the colon in intact form, potentially enabling direct interaction with cancer cells. Interestingly, we found no direct correlation between total phenolic content and antiproliferative activity. In vitro cell cycle analysis revealed that mini purple carrot extracts induced S-phase arrest similar to the chemotherapeutic agent 5-FU, whereas other extracts caused G0/G1-phase arrest. The specific polyphenolic composition appears to be fundamentally important for bioactivity, with chlorogenic acid and diferulic acid-derivative isomer 2 potentially acting synergistically. These findings highlight the importance of carrot biodiversity in delivering functional foods with enhanced health-promoting properties, particularly for colorectal cancer prevention.

## 1. Introduction

Colon cancer is a critical health issue for all generations, regardless of social status and/or income, it is currently the 3rd most common cancer worldwide and is expected to reach 3.2 million new cases in 2040 [1]. Many risk factors contribute to the incidence of colon cancer, including age, race, sex, concomitant inflammatory bowel disease, and genetic predisposition, which is responsible for 35% of cases. The remainder is attributed mainly to epigenetic changes induced by modifiable risk factors, with diet, physical inactivity, alcohol consumption, and smoking being the most critical lifestyle-related factors [2].

In specific cases, diet can directly influence the risk of colon cancer through dietary components or indirectly through the gut microbiome [3]. In general, a high intake of insoluble fibre, fruit and vegetables, and dairy products is characteristic of a healthy diet. There is strong evidence that intake of more than 20 g/day of fibre is associated with a 25% reduction in colon cancer risk. Furthermore, the beneficial effects of fruits and vegetables are attributable to the numerous potentially protective compounds they contain, which modulate biochemical pathways implicated in this disease [4].

Among these protective compounds, polyphenols represent one of the most abundant and diverse groups of phytochemicals in plant-based foods, encompassing thousands of compounds characterised by multiple hydroxyl groups attached to aromatic rings. These bioactive compounds exert pleiotropic health benefits through multiple mechanisms, including direct antioxidant activity, modulation of cellular signalling pathways, anti-inflammatory effects, and influence on gut microbiota composition and metabolism [5]. Epidemiological studies consistently demonstrate inverse associations between dietary polyphenol intake and chronic disease risk, with particularly strong evidence for colorectal cancer prevention.

The health-promoting properties of polyphenols extend beyond cancer prevention to cardiovascular protection, neuroprotection, and metabolic regulation [6,7,8]. Importantly, the bioactivity of polyphenols depends not only on their total concentration but also on their specific structural features, including degree of polymerization, glycosylation patterns, and acylation, which influence their bioavailability, metabolism, and interactions with cellular targets. This structural diversity translates into functional diversity, with different polyphenolic compounds exhibiting complementary and sometimes synergistic biological activities.

Recent epidemiological studies have shown that moderate consumption of carrots is associated with a lower incidence of colorectal cancer, likely due to α- and β-carotene intake [9,10]. Other studies have also demonstrated the protective role of carrots, as this root is the main dietary source of falcarinols and can inhibit growth in human cancer cell lines and neoplastic changes in the colon of rats [11,12]. Likewise, an anthocyanin-rich extract from purple carrot inhibited HT-29 cell growth, underscoring the importance of aglycone glycosylation as a modulator of bioactivity [13].

In addition to carotenoids and polyacetylenes, carrots are among the most important vegetables rich in bioactive compounds, such as phenolic acids and dietary fibre, which have significant health-promoting properties [14,15]. These phytochemicals exert direct antioxidant effects and modulate several metabolic pathways, including cytochrome P450 and signalling pathways mediated by MAP kinases, PI3 kinases, IGF-1, NF-κB, and ROS, which are implicated in both normal and pathological cellular function in organisms [16].

Several factors, including genetics, environmental conditions, and post-harvest storage, influence the profiles and contents of bioactive compounds in carrots, which root colour being one of the most important determinants [17]. While most consumers in the Western world recognise carrots solely as orange vegetables, Eastern culinary traditions embrace a diverse spectrum of purple, yellow, and white varieties, each with distinct phytochemical profiles. For example, orange carrots contain high levels of α- and β-carotene, whereas purple carrots are rich in anthocyanins and other polyphenols. White varieties are abundant in vanillic acid, yellow carrots contain significant amounts of ferulic acid, and both orange and purple carrots contain high concentrations of caffeic and chlorogenic acid derivatives [18]. In recent decades, the scientific community has emphasised the importance of plant food biodiversity and the consumption of foods with distinct phytochemical profiles to maximise health benefits for the population. This has led to the development of various genetic and phenotypic selections aimed at enhancing beneficial compound profiles [19]. A notable example emerged at the beginning of the 21st century with the development of an orange-purple-red carrot variety containing exceptionally high levels of carotenoids, lycopene, and anthocyanins [20].

Beyond colour, carrot varieties of different sizes, determined by the interaction of cultivar genotypic traits, environmental factors, and cultivation techniques, also exhibit significant variation in their phytochemical profiles [21]. Research has demonstrated that the contents of falcarindiol and falcarindiol-3-acetate decrease significantly with increasing root size, whereas root size has no significant effect on α- and β-carotene concentrations [22]. Interestingly, contrasting patterns in phenolic content have been observed: in orange and purple carrots, phenolic compounds increased with carrot size, whereas in yellow carrots they decreased with carrot size [23].

The potential of diverse plant sources as functional foods depends largely on their polyphenolic composition and bioactivity profiles. While grape skins have been extensively studied as a functional food ingredient, with total polyphenolic content ranging from 144 to 298 mg gallic acid equivalents (GAE)/g dry weight and rich in anthocyanins, flavanols, and proanthocyanidins [24,25], carrots offer a distinct and complementary phytochemical profile. Unlike the flavanol-rich composition of grape products, carrots are characterised by hydroxycinnamic acid derivatives, particularly chlorogenic, caffeic, and ferulic acids. Purple carrots uniquely combine substantial anthocyanin content with high levels of phenolic acids, creating a dual polyphenolic composition that may offer synergistic health benefits. Furthermore, the specific polyphenolic composition of carrots, characterised by compounds that exhibit relatively low bioaccessibility during upper gastrointestinal digestion, may be particularly advantageous for colorectal cancer prevention, as these bioactive compounds can reach the colon in more intact forms where they may exert direct protective effects. Additionally, carrots represent a more accessible and culturally acceptable dietary source across diverse populations compared to wine-derived grape products.

Promoting the consumption of non-traditional carrot varieties of diverse colours and sizes requires compelling evidence of their enhanced health benefits. This study addresses this knowledge gap by investigating how plant food biodiversity contributes to human health outcomes. Our study comprehensively evaluated multiple carrot varieties to determine their polyphenolic composition, bioaccessibility following simulated digestion, and potential chemopreventive activity against the HCT-116 colon cancer cell line, a validated early-stage model for colorectal cancer research.

## 2. Materials and Methods

### 2.1. Chemicals and Reagents

α-Amylase (EC 3.2.1.1) and pepsin (EC 3.4.23.1) from porcine gastric mucosa, pancreatin from porcine pancreas, porcine bile extract, MTT reagent (3-(4,5-dimethylthiazol-2-yl)-2,5-diphenyl-2H-tetrazolium bromide), trypan blue solution, dimethyl sulfoxide (DMSO), propidium iodide (PI), Triton X-100, EDTA, RNase-A, 5-Fluorouracil (5-FU), and the standards of bioactive compounds were purchased from Sigma-Aldrich (Madrid, Spain). The cell culture reagents: McCoy’s 5A medium, foetal bovine serum (FBS), penicillin/streptomycin (P/S) solution, trypsin 0.25% in phosphate-buffered saline (PBS) w/o Calcium and w/o magnesium, with w/phenol red and PBS w/o calcium and w/o magnesium were purchased from Gibco (Madrid, Spain).

### 2.2. Plant Materials

**Figure 1:**
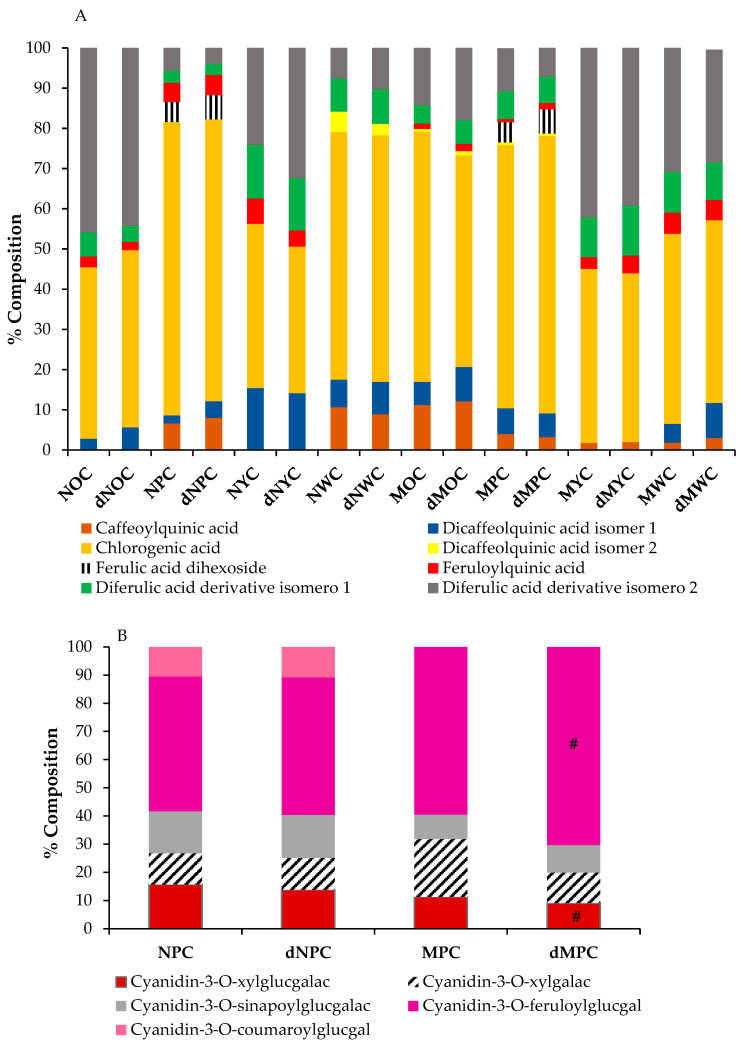
Polyphenols composition from the different colour and size carrots after in vitro digestion assay: (**A**) Phenolic acids, (**B**) Anthocyanins. There is no significant difference in the results for the raw extracts and digested extracts, except for #. Significance: (*p* < 0.5).

Carrots were purchased from Fusion Gusto (Dabrowa, Poland) in June, 2020. The samples were categorised by size and colour. ‘Normal size’ carrots were defined as having diameters between 20 mm and 45 mm (20 mm < d < 45 mm) with weights ranging from 50 g to 150 g (50 g < m < 150 g). ‘Mini size’ carrots had diameters between 10 mm and 20 mm (10 mm < d < 20 mm) and weights between 8 g and 50 g (8 g < m < 50 g) (Figure 1). All carrot samples were freeze-dried for 24 h (Christ Alpha 1–4 LSC, Melsungen, Germany) and subsequently ground into a homogeneous powder using a laboratory mill (IKA A 11, Staufen, Germany) for further analyses.

Our study examined eight distinct carrot varieties categorised by colour and size: normal purple carrot (NPC), normal yellow carrot (NYC), normal orange carrot (NOC), normal white carrot (NWC), mini purple carrot (MiPC), mini yellow carrot (MiYC), mini white carrot (MiWC), and mini orange carrot (MiOC).

### 2.3. Preparation of Polyphenolic Extracts

For polyphenolic extraction, 1.0 ± 0.01 g of each freeze-dried carrot sample was precisely weighed and mixed with 100 mL of methanol. The mixtures were sonicated for 15 min using an ultrasonic cleaner (Model 30, Shenzhen Jietai Ultrasonic Cleaning Equipment Co., Ltd., Shenzhen, China; 6 L capacity, 180 W ultrasonic power, 40 kHz frequency). Subsequently, the extracts were stored at 4 °C for 24 h, after which they were sonicated for a second 15 min period under identical conditions. The extracts were then filtered through a Whatman No. 1 filter paper. All extractions were performed in duplicates.

### 2.4. In Vitro Digestion of Polyphenolic Extracts

In vitro digestion was conducted according to the method described by Jaramillo et al. [26]. Briefly, the digestion process through the mouth, stomach, and small intestine was simulated using appropriate enzymes, with the control groups (without enzymes) running in parallel. The dried methanolic carrot extracts were homogenised with 5 mL of saliva-like solution containing α-amylase and incubated in a water bath with agitation at 37 °C for 5 min. To simulate gastric physiological conditions, samples were acidified to pH 2.0 with HCl, pepsin (200 mg in 5 mL of 0.1 M HCl) was added, and the mixture was incubated with agitation at 37 °C for 1 h (gastric-like digestion). Subsequently, the pH was adjusted to 5.5–6.0 with 1 M NaHCO_3_, followed by the addition of pancreatin (4 mg/mL) and bile extract (25 mg/mL). This mixture was further incubated with agitation for 2 h at 37 °C (small intestinal-like digestion), and enzymatic activity was terminated by cooling on ice at −20 °C for 10 min. Post-digestion solutions were centrifuged at 10,000× *g* for 10 min at 4 °C using a Digtor 22 R centrifuge (ORTOALRESA, Madrid, Spain), filtered, and stored at −80 °C until analysis. All digestion experiments were performed in triplicate.

### 2.5. Analysis and Quantification of Polyphenol Compounds by HPLC/DAD/MS

Polyphenol compounds were analysed using a Jasco-LC-Net II ADC liquid chromatography system equipped with a diode array detector (DAD) (Jasco España, Madrid, España). Separation was performed on a MEDITERRANEA SEA C18 reverse-phase analytical column (25 cm × 4.6 mm i.d., 5 μm particle size; Teknokroma, Barcelona, Spain). The mobile phase consisted of solvent A (water with 1% formic acid) and solvent B (acetonitrile with 1% formic acid), following a gradient programme as follows: solvent B was increased from 0% to 20% over the first 20 min, then gradually to 21% over the next 8 min, held at 21% for 2 min, increased to 30% over the following 10 min, then to 100% over the next 5 min, maintained at 100% for 5 min, and finally returned to the initial conditions over the last 5 min. The flow rate was set at 1 mL/min, and the column temperature was maintained at 30 °C.

Mass spectrometric data were acquired using a Waters 2695 separation module equipped with a Waters 996 PDA detector, connected in series with a single quadrupole mass detector (ACQUITY QDa II, Waters Corporation, Milford, MA, USA). The chromatographic conditions used in this system were identical to those applied in the Jasco setup. Electrospray ionisation (ESI) mass spectra were recorded at ionisation energies of 20 and 50 eV in both negative- and positive-ion modes, with MS scans ranging from *m*/*z* 100 to 1250. The capillary voltage was set to 0.8 kV, the desolvation gas temperature to 200 °C, the source temperature to 100 °C, and the extractor voltage to 12 V.

Spectra from all peaks were recorded in the 200–600 nm range, and the chromatograms were acquired at 280 nm for phenolic acids and 520 nm for anthocyanins. Polyphenol quantification was performed using external calibration curves and reference standards. All analyses were performed in triplicate.

### 2.6. Cell Culture

The human colon adenocarcinoma cell line HCT-116 was obtained from American Type Culture Collection (ATCC) (#CCL 247; Bethesda, MD, USA). Cells were grown at 37 °C with 5% CO_2_ and 90% relative humidity in McCoy’s 5A medium supplemented with 10% heat-inactivated foetal bovine serum, 100 U/mL penicillin, and 100 mg/mL streptomycin.

### 2.7. Cell Viability Assay

Cell viability was determined based on the ability of live cells to reduce MTT [26]. HCT-116 cells were cultured in 96-well plates at a density of 104 cells/well in 200 µL medium. The cells were grown to 70–80% confluence, and then they were treated with different concentrations of carrot polyphenolics (from 0 to 500 μg/mL) and with solvent (DMSO, <0.1%) for 24 and 48 h before adding the MTT (5 mg/mL) solution for 3 h at 37 °C. The culture medium containing MTT was discarded, and 100 μL DMSO was added to the wells, which were incubated in the dark at room temperature for 30 min. The optical density was measured at 570 nm using a microplate reader (MultiskanSpectrum, Thermo Labsystems, Waltham, MA, USA). All MTT assays were performed in three separate trials, and each set was applied in six replicates.

### 2.8. Cell-Cycle Profiling

For cell cycle profiling, flow cytometry analysis was performed as described previously [26]. Briefly, HCT-116 colorectal cancer cells were cultured in a 6-well plate at 2.5 × 105 cells per well in McCoy’s 5A medium and treated with different concentrations of carrot polyphenolics in a dose-dependent manner for the indicated time. After treatment, cells were harvested from the plate using 0.05% trypsin-EDTA and gently washed twice with 1× PBS. Washed cells were fixed in 70% ice-cold ethanol and stored at −80 °C until analysis. For cell cycle analysis, cells were thawed, washed twice with PBS, and suspended in 0.5 mL of staining reagent (50 mg/mL PI, 50 U/mL RNase, 0.1 mM EDTA, 0.1% Triton X-100, PBS). After incubation for 30 min at 37 °C in the dark, DNA fluorescence was measured using a Becton Dickinson (BD Biosciences, Madrid, Spain) FACScanto II flow cytometer at an excitation wavelength of 488 nm and emission wavelength of 585 nm. Pulse-width area signals were used to discriminate between G2 cells and cell doublets. Data were analysed using FlowJo v10.7.1 software (Tree Star, Ashland, OR, USA). Approximately 10,000 events per sample were analysed, and the relative distribution of cells in each phase (G0/G1, S, and G2/M) was displayed as a histogram.

### 2.9. Statistical Analysis

The results were analysed using IBM^®^ SPSS^®^ statistics v23.0, for Windows (IBM, Madrid, Spain). All quantitative data are represented as the mean ± SD from triplicate experiments performed in parallel, unless otherwise indicated. Mean values among treatment groups were compared using analysis of variance (ANOVA) followed by Duncan’s multiple comparison test. Statistical significance was set at *p* < 0.05.

## 3. Results

In this study, eight different carrot extracts, orange, purple, yellow, and white, with two different sizes (standard and mini sizes), were used to analyse the polyphenolic content, bioaccessibility, and the relationship between their anti-proliferative effects on colon cancer cell lines (HCT-166) to increase consumer attention to healthier carrots.

### 3.1. Phytochemical Composition

Eight phenolic acids, primarily derivatives of caffeic and ferulic acid, were detected in all the carrot samples analysed. Chlorogenic acid was identified as the predominant phenolic compound in all samples, consistent with previous reports [23,27,28], followed by a ferulic acid derivative (mainly diferulic acid derivative isomer 2), and caffeoylquinic acid (Table 1).

As expected, the purple carrots showed the highest polyphenolic content, as they contained not only phenolic compounds, including chlorogenic, caffeic, and ferulic acids and their derivatives, but also anthocyanins (representing approximately 35% of the total content). At the same time, the other samples lacked these pigments, with carotenoids responsible for the colour of the orange and yellow samples.

Surprisingly, carrot size emerged as a determinant of polyphenolic content, with miniature varieties consistently exhibiting higher polyphenol concentrations than their normal-sized counterparts, regardless of colour. The decreasing trend in total polyphenol content followed the order: mini purple (MPC) > normal purple (NPC) > mini orange (MOC) > normal orange (NOC) > mini yellow (MYC) > mini white (MWC) = normal white (NWC) > standard yellow (NYC) (Table 1). This relative distribution of total phenolics across carrot varieties of different colours aligns with patterns observed in previous studies [29]. Remarkably, in our study, white carrots exhibited total polyphenol content comparable to that of yellow varieties, with no significant size-related differences. This finding contrasts with previous research [30], which reported that white carrots typically have the lowest phenolic levels, approximately half the concentration of yellow varieties. Overall, our results indicated that carrot size significantly influenced phytochemical composition, consistent with previous findings in carrots [23] and other fruits and vegetables [31], where smaller specimens generally exhibited higher polyphenolic content. When examining the specific phenolic acid profiles, we did not identify a consistent pattern attributable solely to carrot colour or size. However, we observed distinct compositional similarities between certain groupings: normal white carrots (NWC), mini purple carrots (MPC), and mini orange carrots (MOC) shared comparable phenolic acid profiles, while normal orange carrots (NOC) and mini yellow carrots (MYC) formed another group with similar compositions.

Chromatographic analysis of the anthocyanin profiles revealed five distinct anthocyanins, all cyanidin-based compounds identified as: cyanidin-3-O-xylosyl-glucosylgalactoside, cyanidin-3-O-xylosylgalactoside, and three acylated derivatives of the triglycoside (with sinapoyl, feruloyl, and p-coumaroyl moieties), cyanidin-3-O-xylosyl-sinapoyl-glucosylgalactoside, cyanidin-3-O-xylosyl-feruloyl-glucosylgalactoside, and cyanidin-3-O-xylosyl-p-coumaroylglucosyl-galactoside (Table 1). Feruloyl-acylated cyanidin glycoside was the most abundant compound in both purple carrot varieties. This predominance of cyanidin-3-O-xylosyl-feruloyl-glucosylgalactoside aligns with previous findings [32], although other researchers have reported cyanidin-3-sinapoylxylosyl-glucose as the primary pigment [33]. The total anthocyanin concentrations in both purple carrot varieties were comparable and consistent with those reported by Blando et al. [34]. Notably, the anthocyanin content was higher in normal-sized purple carrots than in miniature carrots, representing 50% and 25% of the total polyphenolic content, respectively. This size-related difference is consistent with several studies reporting higher anthocyanin concentrations in larger carrots [23], although contradictory findings have also been reported [35].

Interestingly, mini purple carrots exhibited a distinct anthocyanin profile, with cyanidin-3-O-xylosyl-feruloyl-glucosylgalactoside predominating (60% of the total anthocyanin content), followed by cyanidin-3-O-xylosylgalactoside (20%), cyanidin-3-O-xylosyl-glucosylgalactoside (11%), and cyanidin-3-O-xylosyl-sinapoyl-glucosylgalactoside (9%). In normal-sized purple carrots (NPC), cyanidin-3-O-xylosyl-feruloyl-glucosylgalactoside remained the most abundant anthocyanin. Still, at a lower proportion (48%), while cyanidin-3-O-xylosyl-glucosylgalactoside and cyanidin-3-O-xylosyl-sinapoyl-glucosylgalactoside were present at 16% and 15%, respectively, and cyanidin-3-O-xylosylgalactoside and cyanidin-3-O-xylosyl-p-coumaroylglucosyl-galactoside constituted 11% of the anthocyanin content (Table 1).

### 3.2. Bioaccessibility

To know the fraction of the polyphenolic dietary intake from carrots that could reach the colon and exert their biological activity, polyphenolic extracts were exposed to in vitro digestion. Our results showed that the phenolic acids, as well as anthocyanins, were stable (between 92 and 97% final recovery) during the different phases of the simulated digestion (Figure 1).

Bioaccessibility, defined as the fraction of ingested compounds released from the food matrix during gastrointestinal digestion and available for intestinal absorption [36], was exceptionally high for all the carrot samples analysed. These findings align with previous research [37], although contrasting results exist in the literature, with some researchers reporting phenolic bioaccessibility as low as 13% for normal orange carrots (NOC) [38]. Despite previous studies documenting substantial decreases in anthocyanin concentrations during oral digestion of black carrots [39] and during the intestinal phase [40], our in vitro digestion experiments revealed no significant differences in anthocyanin stability throughout the digestive process.

**Figure 2:**
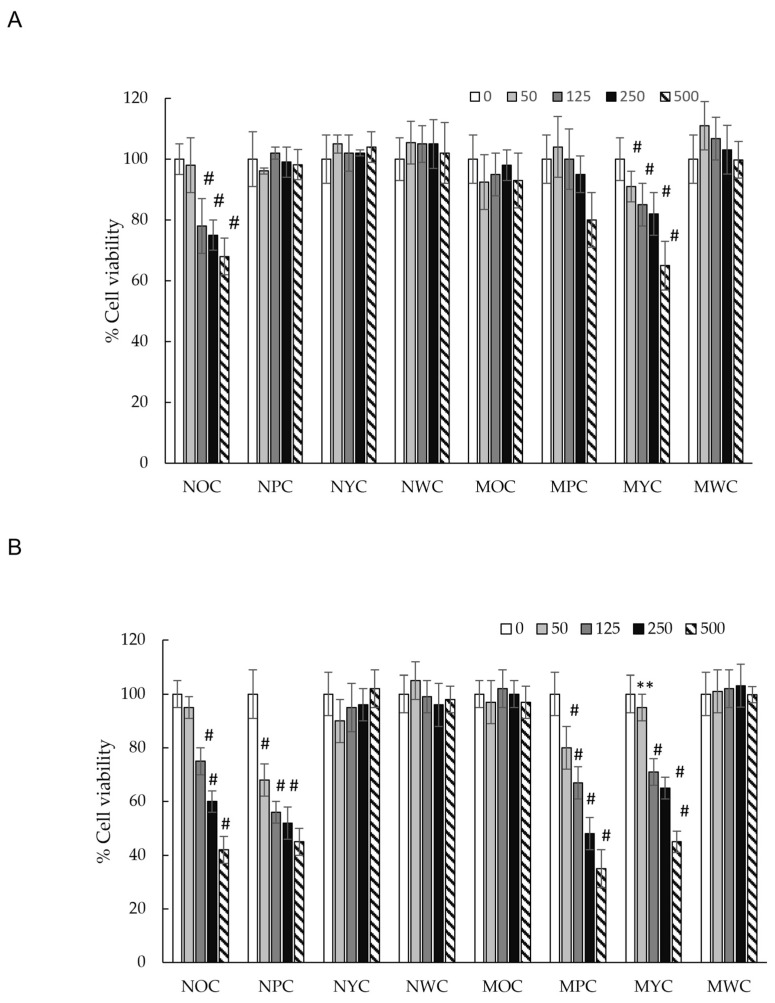
Effect of different carrot polyphenolic concentrations on cell viability (MTT assay) on HCT-116 cells incubated for 24 h (**A**) and 48 h (**B**). There is a significant difference in the results for the extract’s effect. Significance: # (*p* < 0.05), ** (*p* < 0.01) vs. McCoy’s 5A.

The chemical structure is one of the most critical determinants of the bioaccessibility of bioactive compounds, with acylated cyanidins exhibiting greater chemical stability during digestion than non-acylated cyanidins [41]. In our carrot samples, the predominant anthocyanin was cyanidin-3-O-xylosyl-feruloyl-glucosylgalactoside, a ferulic acid-acylated cyanidin derivative, accounting for 48% of total anthocyanin content in NPC and 60% in MPC (Figure 2B). This composition likely accounts for the high stability observed in our study, consistent with findings reported by other researchers [37,42]. The anthocyanin profiles remained essentially unchanged following the simulated digestion process across most samples, with one notable exception: in mini purple carrots (MPC), cyanidin-3-O-xylosylgalactoside decreased by 50%, accompanied by a corresponding increase in cyanidin-3-O-xylosyl-feruloyl-glucosylgalactoside. These results contrast with those of previous studies of black carrots with similar anthocyanin compositions, in which anthocyanins were not detected after in vitro digestion [43].

### 3.3. Cytotoxic Effect of the Different Polyphenol Extracts on the Human Colorectal Carcinoma Cell Line HCT-116

To evaluate the cytotoxic effects of phenolic extracts from different carrot varieties, HCT-116 cells were treated with increasing concentrations of the extract (50, 125, 250, and 500 µg/mL) and monitored at 24 and 48 h. As shown in Figure 2, at the 24 h time point, only the normal orange carrot (NOC) and mini yellow carrot (MYC) extracts demonstrated modest cytotoxicity (~40% growth inhibition at the highest concentration tested). In contrast, all other extracts showed no significant effect on cell viability. However, after 48 h of exposure, we observed remarkable inhibition of HCT-116 cell growth in a time-dependent manner for mini purple carrot (MPC), normal orange carrot (NOC), mini yellow carrot (MYC), and normal purple carrot (NPC) extracts, each achieving approximately 70% reduction in cell viability compared to untreated controls. The remaining carrot varieties showed no detectable cytotoxicity, even at extended time points.

Previous study research demonstrated that purple carrot polyphenol extract exhibited moderate cytotoxicity (12% inhibition) against the HCT-116 cell line after 24 h of incubation at 1 mg/mL [44]. In contrast, our results showed no cytotoxicity of normal purple carrot extract at 0.5 mg/mL after 24 h. However, after 48 h of incubation, dose-dependent cytotoxicity became evident, with cell viability decreasing at concentrations ranging from 125 µg/mL (44% inhibition) to 500 µg/mL (55% inhibition), indicating a more pronounced time-dependent effect. Notably, the mini purple carrot (MPC) extract demonstrated significantly higher cytotoxic activity after 48 h (IC50 = 315 µg/mL), which may be attributed to its elevated phenolic acid content, approximately twice that of the normal purple carrot (NPC) extract (Table 1).

**Figure 3:**
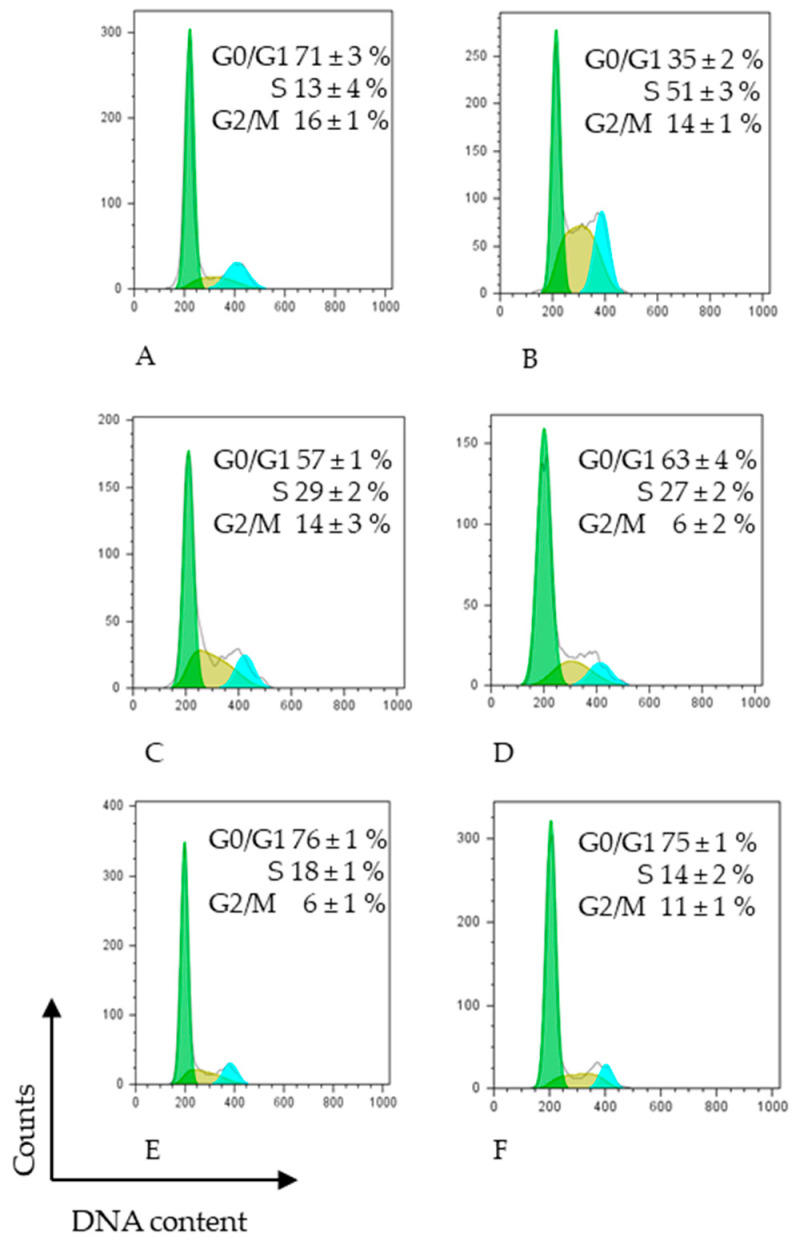
Effect of different extracts on cell cycle distribution of HCT-116 human colon cancer cells assessed by FACS analysis. HCT-116 cells were treated with 5-FU (DMSO) or IC50 (μg/mL) of different carrots extracts in the presence of complete medium for 48 h: (**A**) untreated cells, (**B**) 100 ng/mL 5-FU, (**C**) 300 μg/mL MPC, (**D**) 400 μg/mL NPC, (**E**) 400 μg/mL NOC, (**F**) 400 μg/mL MYC, and then subjected to FACS cell cycle analysis as described in Section 2. The data represent the average median of at least three independent experiments SD. *p* < 0.05 was determined by one-way analysis of variance followed by Duncan’s test. Different color represents different cell cycle phases.

In brief, no statistically significant differences in cytotoxic activity were observed among the normal purple carrot (NPC), normal orange carrot (NOC), and mini yellow carrot (MYC) extracts (IC50 ~395 µg/mL). Interestingly, despite having a higher total polyphenol content than the aforementioned varieties (Table 1), the mini orange carrot (MOC) extract exhibited no detectable cytotoxicity. This observation indicates the absence of a direct correlation between total polyphenol content and cytotoxic activity. However, significant differences in the phenolic composition of the samples were observed. Specifically, the phenolic profiles of NOC and MYC extracts were characterised by approximately equal proportions of chlorogenic acid and the diferulic acid derivative isomer 2 (42% and 46% of the total phenolic content, respectively), a pattern distinctly different from that of other samples (Figure 3). This particular compositional balance may be crucial for the observed cytotoxic effects of these extracts relative to MOC, potentially enabling synergistic interactions among these compounds that are independent of total phenolic content.

### 3.4. Cell Cycle Effect of the Different Polyphenol Extracts on the Human Colorectal Carcinoma Cell Line HCT-116

The significant antiproliferative activity observed in extracts of mini purple carrot (MPC), normal purple carrot (NPC), normal orange carrot (NOC), and mini yellow carrot (MYC) warrants further investigation into their mechanisms of action. Therefore, we conducted cell cycle analyses on the HCT-116 cell line following 48 h of treatment with each extract at its respective IC50 concentration. 5-fluorouracil (5-FU) served as a positive control, given its established use as a chemotherapeutic agent in metastatic colorectal cancer and its known mechanism of action as a topoisomerase I inhibitor [45].

Figure 3 illustrates the percentage distribution of cells in each phase of the HCT-116 cell cycle, following treatment. The results demonstrated that cells treated with 5-FU were predominantly arrested in S phase. Similarly, treatment with the MPC extract at 310 µg/mL doubled the proportion of HCT-116 cells in the S phase after 48 h compared with untreated controls, accompanied by a corresponding decrease in the G0/G1 phase. The NPC extract induced a different cell distribution pattern, characterised by a moderate decrease in the G0/G1 phase relative to untreated cells, an increase in the S phase (as in the sample (MPC)), and a marked reduction in the G2/M phase. NOC extract treatment resulted in comparable proportions of cells in the G0/G1 and S phases, whereas it significantly reduced the G2/M phase compared with both untreated controls and 5-FU treatment. The MYC extract produced a cell cycle distribution pattern similar to that observed with NOC treatment.

Our results demonstrated that the mini purple carrot (MPC) extract exerted a cell cycle effect comparable to that of 5-FU, specifically inducing S-phase arrest. In contrast, the other carrot extracts produced distinct effects on cell cycle distribution, characterised by modest yet statistically significant G0/G1-phase arrest.

## 4. Discussion

In recent years, researchers have focused on providing health-promoting foods that address malnutrition and prevent chronic diseases, including less conventional plant-based foods. Carrots are a valuable source of phytochemicals, primarily comprising four categories: phenolics, carotenoids, polyacetylenes, and ascorbic acid compounds, all of which offer significant health benefits. These root vegetables are classified as ‘vitaminised foods’ because a 100 g serving of raw carrots provides approximately 120% of the recommended daily allowance (RDA) of vitamin A, 5% of vitamin C, and 4.5% of vitamin E [46]. Several factors influence the composition and concentration of these phytochemicals, with phenotype among the most significant determinants [14].

Currently, carrot varieties exhibit substantial biodiversity, with variations in size, shape, colour, flavour, and production characteristics. Although consumers in Western countries predominantly associate carrots with orange colouration, Eastern markets feature a broader spectrum of varieties, including purple, yellow, and white. A recent cross-cultural study examining carrot colour preferences among European, Asian, and South American consumers found that carrots with non-traditional colours were rated significantly lower than orange carrots in terms of perceived healthiness, attractiveness, purchase intention, familiarity, and similarity, with white carrots receiving the lowest ratings, regardless of their actual phytochemical content [47].

For instance, a previous study showed that although purple carrots contain ten-fold higher levels of phenolic compounds than yellow and white varieties and five-fold more than orange carrots, their compositional profile remains similar, with 5-caffeoylquinic acid as the predominant compound [28]. Comparable findings emerged from an analysis of 12 carrot varieties with different colours and sizes, revealing that phenolic acid content increased in the following sequence: yellow > white > orange > purple. Size proved to be a critical factor, as varieties with the lowest phenolic content showed significant increases when examined in smaller sizes [23]. Furthermore, substantial evidence supports the relationship between biodiversity and human health benefits [48].

In this study, we compared four carrot cultivars (orange, white, yellow, and purple) of different sizes and analysed their polyphenol content, bioaccessibility, and antiproliferative activity against the HCT-116 colon cancer cell line. The results revealed that vegetable size influenced the phytochemical composition more significantly than colour, with mini purple carrots exhibiting exceptionally high polyphenolic content. Notably, the anthocyanin concentration was higher in normal-sized carrots than in mini purple carrots.

To date, limited data exist regarding the impact of gastrointestinal digestion on polyphenol extracts from differently coloured and sized carrots. Our findings indicated that the bioaccessibility of polyphenolic compounds remained unaffected by carrot colour or size. However, it is important to acknowledge that in vitro digestion provides only an approximation of physiological processes, and that our experimental design did not account for the effects of the food matrix. Nonetheless, the low bioaccessibility of carrot polyphenols after simulated digestion suggests that these compounds may reach the colon in an intact chemical form, potentially exerting direct biological effects that could reduce the risk of various metabolic disorders, including cancer [49]. This in vitro research is useful for evaluating the bioaccessibility and initial biological activity of compounds and facilitate the selection of promising variety carrot, in this case MPC.

Regarding the antiproliferative effects on HCT-116 cells, we found that the extracts with the highest cytotoxicity at 48 h were, in descending order: mini purple carrot (MPC), normal purple carrot (NPC), normal orange carrot (NOC), and mini yellow carrot (MYC). Interestingly, we observed no correlation between the total phenolic content and antiproliferative activity. Studies on different grape genotypes showed that there was no correlation between polyphenol content and anti-cancer activity in a human colon cancer cell line [49]. Interestingly, authors pointed out that anthocyanidins showed the strongest positive correlation with the anti-cancer activity of the extracts, similar to purple carrots Additionally, our results indicated that the MPC extract exhibited a cell cycle effect similar to that of 5-FU (a chemotherapeutic agent that primarily functions through S-phase arrest). In contrast, the other extracts demonstrated distinct mechanisms of action.

These findings collectively suggest that the specific compositional profile of these extracts, particularly the similar proportions of chlorogenic acid and diferulic acid derivative isomer 2, representing 43% each of these compounds in both samples, plays a fundamental role in their antiproliferative activity. Chlorogenic acid has been established as a key phenolic compound in medicinal plants that exhibits significant cytotoxicity, induces cell cycle arrest, and promotes apoptosis in colon cancer cell lines [50,51]. Similarly, ferulic acid and its derivatives have demonstrated notable chemoprotective properties in colon cancer cells [52,53]. Therefore, the distinctive compositional balance in normal orange carrot (NOC) and mini yellow carrot (MYC) extracts likely underlies their observed antiproliferative activity, potentially through synergistic interactions among these compounds.

Regarding anthocyanins, both purple carrot samples contained similar total concentrations but differed in their compositional profiles. In normal purple carrot (NPC) extract, cyanidin-3-O-xylosyl-feruloyl-glucosylgalactoside is predominant (48%), with the remaining anthocyanins ranging from 11% to 16%. In the mini-purple carrot (MPC) extract, cyanidin-3-O-xylosyl-feruloyl-glucosylgalactoside represented an even larger fraction (60%), followed by cyanidin-3-O-xylosyl-galactoside, while cyanidin-3-O-xylosyl-p-coumaroylglucosyl-galactoside was notably absent. Multiple studies have attributed the chemopreventive properties of purple carrots primarily to their anthocyanin content [49]. Notably, an anthocyanin-rich extract from purple carrot demonstrated an IC50 (concentration required to inhibit 50% cell proliferation) of 68.5 µg/mL after 48 h in the HT-29 advanced human colon cancer cell line, with researchers suggesting that specific acylation and glycosylation patterns of anthocyanins significantly influence their biological activities [40]. Although both purple carrot samples contained equivalent amounts of nonacylated anthocyanins (Table 1), previous studies have established that these nonacylated forms are more potent inhibitors of colon cancer cells than their acylated counterparts. This suggests that the higher proportion of cyanidin-3-O-xylosyl-galactoside in the MPC extract accounts for its enhanced cytotoxic activity, or that differences in mono-acylated anthocyanin composition between the samples play a crucial role in the observed biological effects.

Further investigations are necessary to thoroughly evaluate the potential synergistic and/or antagonistic interactions between these compounds and their contributions to the observed biological activities. Such research would facilitate the selection and development of new carrot varieties with enhanced health-promoting properties, potentially increasing consumer acceptance and consumption of nontraditional carrot varieties. It would be interesting to carry out in vivo assays, in order to determine systemic and local effects that cannot be fully reproduced in vitro due to they are essential for confirming biological relevance and health impact under real physiological conditions.

## 5. Conclusions

Our comprehensive investigation of carrot biodiversity has significant implications for both nutritional science and functional food development. Mini purple carrots have emerged as exceptional candidates for health-promoting applications, exhibiting the highest polyphenolic content and demonstrating superior antiproliferative activity against colon cancer cells. Mini purple carrots exhibited the highest total polyphenolic content (2973.5 ± 39.8 mg/100 g DW), with chlorogenic acid as the predominant phenolic acid. The observed low bioaccessibility of polyphenolic compounds in our in vitro simulated digestion model, rather than being a limitation, may represent an advantage, allowing these bioactive compounds to reach the colon with their structures intact, where they can directly interact with colonic tissues.

The distinctive finding that specific compositional profiles of phenolic compounds, particularly balanced proportions of chlorogenic acid and diferulic acid derivatives, (approximately 43–46% of total phenolic content in bioactive extracts), appear more critical for bioactivity than total phenolic content, challenges the conventional understanding. Furthermore, our cell cycle analysis revealed that mini-purple carrot extracts induced S-phase arrest similar to that induced by 5-FU, suggesting potential applications in cancer prevention strategies.

These results highlight the untapped potential of non-traditional carrot varieties as functional food ingredients or dietary supplements that target colorectal health. Our findings underscore the importance of considering both size and colour when selecting carrot cultivars for nutritional interventions or breeding programmes. The superior properties of miniature varieties, particularly mini purple carrots, represent an opportunity to develop novel high-value food products with enhanced health benefits.

This study was designed as an initial evaluation of the potential anticancer activity of carrot extracts using a single cancer cell line model. While this approach provides valuable preliminary insights, it does not capture the heterogeneity of cancer or confirm selectivity against non-tumorigenic cells. Future research should include multiple cancer cell lines and normal cells to validate the generalizability and specificity of the observed effects.

Future research should also focus on translating these findings into in vivo models and human trials, exploring synergistic interactions between specific polyphenolic compounds, and developing consumer-acceptable food products that maximise the delivery of these bioactive compounds to target tissues. Ultimately, this study contributes to the growing body of evidence supporting the cultivation and consumption of diverse plant foods to optimise health outcomes, particularly for colorectal cancer prevention.

## Figures and Tables

**Table 1:** The content of polyphenols (mg/100 g^−1^ DW) in carrot extracts.

Compound	Normal Orange	Normal Purple	Normal Yellow	Normal White	Mini Orange	Mini Purple	Mini Yellow	Mini White
Caffeoylquinic acid	nd	59.1 ± 3.2 bf	nd	30.0 ± 1.3 cb	130.4 ± 5.1 ac	191.0 ± 8.7 ac	11.4 ± 0.1 dd	5.4 ± 0.1 ef
3,5-Dicaffeolquinic acid isomer 1	35.8 ± 0.1 cb	17.6 ± 0.3 fi	28.3 ± 1.5 dc	19.3 ± 0.2 ed	66.9 ± 4.2 bd	142.5 ± 6.2 ae	nd	13.7 ± 0.3 ge
Chlorogenic acid	330.9 ± 4.2 da	648.4 ± 9.1 ca	75.1 ± 7.2 ha	173.5 ± 5.2 fa	723.7 ± 16.2 ba	1455.2 ± 23.2 aa	278.3 ± 6.4 ea	139.0 ± 3.6 ga
3,5-Dicaffeoylquinic acid isomer 2	nd	nd	nd	13.9 ± 0.4 ae	8.6 ± 0.2 cg	13.8 ± 0.2 bi	nd	nd
Ferulic acid dihexoside	nd	44.1 ± 2.5 ag	nd	nd	nd	10.7 ± 0.2 bj	nd	nd
Feruloylquinic acid	15.0 ± 0.1 dc	42.7 ± 3.0 ag	11.5 ± 1.2 ed	nd	15.1 ± 0.8 df	17.1 ± 0.6 ch	18.7 ± 0.2 bc	15.4 ± 0.1 dd
Diferulic acid derivative isomer 1	36.0 ± 0.5 db	26.4 ± 1.8 fh	24.5 ± 1.6 fc	23.3 ± 1.1 fc	52.6 ± 3.2 ce	155.2 ± 6.2 ad	63.2 ± 1.5 bb	29.7 ± 1.4 ec
Diferulic acid derivative isomer 2	320.7 ± 6.3 aa	50.5 ± 4.2 ef	44.2 ± 2.6 eb	21.3 ± 0.9 fc	166 ± 9.4 cb	237.6 ± 8.2 cb	270.6 ± 10.3 ba	90.5 ± 3.6 db
**Total phenolic acids**	**738 ± 11.2 d**	**888.8 ± 24.1 c**	**183.6 ± 14.1 g**	**281.3 ± 9.1 f**	**1163.8 ± 39.1 b**	**2223.0 ± 53.5 a**	**642.2 ± 18.5 e**	**293.8 ± 9.1 f**
Cyanidin-3-O-xylosyl-glucosylgalactoside		139.6 ± 3.4 ac				85.0 ± 6.7 bf		
Cyanidin-3-O-xylosyl-galactoside		98.6 ± 2.8 bd				153.7 ± 6.2 ad		
Cyanidin-3-O-xylosyl-sinapoyl-glucosylgalactoside		132.1 ± 5.2 ac				65.0 ± 4.8 bg		
Cyanidin-3-O-xylosyl-feruloyl-glucosylgalactoside		424.8 ± 8.1 bb				446.8 ± 9.1 ab		
Cyanidin-3-O-xylosyl-p-coumaroylglucosyl-galactoside		93.6 ± 1.4 ae				nd		
**Total anthocyanins**		**888.8 ± 20.6 a**				**750.5 ± 13.0 a**		
**Total polyphenolic content**	**738.3 ± 11.2 d**	**1777.6 ± 44.7 b**	**183.6 ± 14.1 g**	**281.3 ± 9.1 f**	**1163.8 ± 39.1 c**	**2973.5 ± 39.8 a**	**642.2 ± 18.5 e**	**293.8 ± 9.1 f**

Values represent the mean ± standard error of three replicates. In each file and column, values with different letters are significantly different (*p* < 0.05). nd, not detected.

## Data Availability

The original contributions presented in this study are included in the article. Further inquiries can be directed to the corresponding authors.
